# Incidence of Total Stroke, Stroke Subtypes, and Myocardial Infarction in the Japanese Population: The JMS Cohort Study

**DOI:** 10.2188/jea.JE2007438

**Published:** 2008-08-07

**Authors:** Shizukiyo Ishikawa, Kazunori Kayaba, Tadao Gotoh, Naoki Nago, Yosikazu Nakamura, Akizumi Tsutsumi, Eiji Kajii, the Jichi Medical School (JMS) Cohort Study Group

**Affiliations:** 1 Division of Community and Family Medicine, Center for Community Medicine, Jichi Medical University; 2 School of Health and Social Services, Saitama Prefectural University; 3 Community Medicine Education Center, Japan Association for Development of Community Medicine; 4 Department of Public Health, Jichi Medical University; 5 University of Occupational and Environmental Health, Japan

**Keywords:** Incidence, Stroke, Myocardial Infarction, Cohort Studies, Asian Continental Ancestry Group

## Abstract

**Background:**

Previous reports indicated that the incidence rate of stroke was higher in Japan than in Western countries, but the converse was true in the case of myocardial infarction (MI). However, few population-based studies on the incidence rates of stroke and MI have been conducted in Japan.

**Methods:**

The Jichi Medical School (JMS) Cohort Study is a multicenter population-based cohort study that was conducted in 12 districts in Japan. Baseline data were collected between April 1992 and July 1995. We examined samples from 4,869 men and 7,519 women, whose mean ages were 55.2 and 55.3 years, respectively. The incidence of stroke, stroke subtypes, and MI were monitored.

**Results:**

The mean follow-up duration was 10.7 years. A total of 229 strokes and 64 MIs occurred in men, and 221 strokes and 28 MIs occurred in women. The age-adjusted incidence rates (per 100,000 person-years) of stroke were 332 and 221 and those of MI were 84 and 31 in men and women, respectively. In the case of both sexes, the incidence rates of stroke and MI were the highest in the group of subjects aged > 70 years.

**Conclusion:**

We reported current data on the incidence rates of stroke and MI in Japan. The incidence rate of stroke remains high, considerably higher than that of MI, in both men and women. The incidence rates of both stroke and MI were higher in men than in women.

## INTRODUCTION

In Japan, the stroke mortality rate has declined significantly during the period from 1965 through 2000. Even after malignancy became the leading cause of death in 1981, stroke and heart disease remained the second and the third leading causes of mortality, respectively in Japan over this period.^1^ Both remain major health problems in Japan as in other developed countries.

It has been reported in previous international comparative studies that the incidence and mortality of stroke were higher, and those of myocardial infarction (MI) were lower in Japan than in Western countries.^2^^,^^3^ Many population-based cohort studies have been conducted in Western countries,^4^^-^^13^ but few cohort studies have been carried out in Japan recently.^14^^,^^15^ However, some studies examining the incidence of stroke and MI commenced several decades ago.^16^^-^^19^

It remains unclear why the mortality and incidence rates of coronary heart disease (CHD) have been lower and those of stroke higher in Japan than in Western countries. In the Ni-Hon-San study, which compared individuals of Japanese ancestry living in Japan, Hawaii, and California 35 years ago, it was confirmed that significant differences existed in the incidence rates of stroke and MI among the 3 populations.^2^ While these differences may be attributable to environmental factors, they were only partially explained by known risk factors such as blood pressure, smoking, and serum cholesterol.

The objective of the present study is to examine the incidence of stroke and MI in a multicenter population-based cohort study, which commenced in the 1990s. We recently finished the follow-up for the incidence of stroke and MI.

## METHODS

### Subjects

The Jichi Medical School (JMS) Cohort Study was conducted to investigate the risk of cardiovascular diseases, stroke, and MI. There were 12,490 Japanese men and women from 12 communities across Japan in the present study. Baseline data were obtained between April 1992 and July 1995. A detailed description about standardized collection of baseline data has been published previously.^20^ In each community, at least 1 alumnus of JMS worked as a physician and played an important role in data collection and collaboration with the local government. Mass screening examinations for cardiovascular diseases have been conducted at these sites since 1983, in accordance with the Health and Medical Service Law for the Aged. This system was used to collect the baseline data for the present study. A municipal government office in each community site sent personal invitations to all the potential participants for the examination by letter or public information. The target subjects were residents aged 40-69 years in 8 communities, those aged 20-69 years in 1 community, and those aged 35 years and older (no upper limit) in 1 community. All adults (no age limit) were examined in the other communities. The participation rate varied in each community (26%-90%), and the overall participation rate of those invited to the mass screening examination program was 65.4%.

### Ethical Issues

The study design and procedure were approved by each community government and by the Ethical Committee of Epidemiologic Research at Jichi Medical University. Written informed consent for the study was obtained individually from those who responded to the mass screening examination health check-up. At visits, participants were informed that data would be obtained from questionnaires and blood samples, and that their health status would be followed up through a review of their hospital medical records if a stroke or MI was suspected to have occurred.

### Follow-up System

The mass screening examination system was used to obtain the baseline data for the cohort study. This system was also used to follow-up the subjects each year. After enrollment in the study, subjects were asked whether they had a history of stroke or cardiovascular diseases. Those with such a history were asked when and which hospital they visited. Subjects who did not attend the screening examination were contacted by mail or phone. Medical records were checked if the subjects were hospitalized for any reason. Public health nurses also visited the homes of the subjects to obtain further information.

If an incident case of stroke or MI was suspected, duplicate computer tomography scans or magnetic resonance images in the case of stroke and electrocardiograms in the case of MI were requested. Death certificates were collected until the end of 2005 from public health centers with the official permission of the Agency of General Affairs and the Ministry of Health, Labour and Welfare. Data on subjects who moved out of the study area were obtained annually from the relevant municipal government.

### Diagnostic Criteria

The diagnoses were carried out independently by a Diagnosis Committee, composed of 1 radiologist, 1 neurologist, and 2 cardiologists. The criteria for stroke were sudden onset of a focal and nonconvulsive neurological deficit that lasted for more than 24 h, and the stroke subtype was determined according to the criteria of the National Institute of Neurological Disorders and Stroke.^21^ MI was diagnosed based on the criteria of the World Health Organization Multinational Monitoring of Trends and Determinants in Cardiovascular Disease (MONICA) Project-a multinational collaborative project that was conducted from the mid-1980s through the mid-1990s for the monitoring of coronary events.^22^

### Statistical Analysis

Data regarding the variables were expressed as mean ± standard deviation (SD), except in the case of triglycerides (TGs). The distribution of TGs was very skewed and was hence expressed as geometric mean ± SD. Data regarding proportions were expressed as percentages. Incidence rates were calculated and expressed in terms of per 100,000 person-years. Direct standardization was conducted to adjust the rates to the age structure of the Japanese population in 1985. While analyzing outcomes, we excluded subjects who reported a positive history at the time of data collection, i.e., subjects with a history of stroke and MI were excluded while calculating the stroke and MI incidence rates, respectively. These analyses were performed using SAS^®^ software version 8.2.

## RESULTS

Among the 12,490 participants, 95 declined follow-up, and 7 subjects could not be contacted after baseline data collection. Thus, a total of 4,869 men and 7,519 women were followed up. The follow-up rate was 99.2%. The mean age at the time of baseline data collection was 55.2 and 55.3 years in the case of men and women, respectively. The mean duration of follow-up was 10.7 years.

The baseline characteristics of the subjects are shown in Table 1. Few subjects had histories of stroke or MI. A total of 450 cases of stroke and 92 cases of MI were confirmed during the follow-up period. Of these, 229 strokes and 64 MIs occurred in men, and 221 strokes and 28 MIs occurred in women.

**Table 1.** Baseline characteristics of the participants of the JMS Cohort Study in Japan.

	Men				Women			
	
	n	Mean	SD	Proportion (%)	n	Mean	SD	Proportion (%)
Age (y)	4,869	55.2	12.0		7,519	55.3	11.4	
Systolic blood pressure (mmHg)	4,665	131.4	20.5		7,283	128.2	21.0	
Diastolic blood pressure (mmHg)	4,665	79.2	12.3		7,283	76.3	12.1	
Total cholesterol (mg/dL)	4,799	184.9	34.1		7,437	196.7	34.8	
Triglycerides (mg/dL)*	4,799	108.8	(63.0-187.9)		7,436	95.6	(57.6-158.5)	
HDL cholesterol (mg/dL)	4,800	48.8	13.4		7,437	52.6	12.5	
Body-mass index (kg/m^2^)	4,649	23.0	2.9		7,243	23.2	3.2	
Smoking status								
Current smoker	2,281			50.4	382			5.5
Ex-smoker	1,282			28.3	195			2.8
Non-smoker	961			21.3	6,363			91.7
Drinking status								
Current drinker	3,307			75.1	1,691			24.9
Ex-drinker	160			3.6	102			1.5
Non-drinker	937			21.3	4,987			73.6
Past history								
Stroke	62			1.3	50			0.7
Myocardial infarction	40			0.8	25			0.3
Medication								
Hypertension	452			9.3	864			11.5
Diabetes mellitus	108			2.2	119			1.6
Hyperlipidemia	57			1.2	146			1.9

SD: Standard deviation

HDL: High-density cholesterol

*: Geometric mean ±SD

The crude annual incidence rates of stroke were 450.8 and 273.1 per 100,000 person-years in men and women, respectively. These rates increased with age in both sexes. The crude incidence rates of cerebral hemorrhage, cerebral infarction, and subarachnoid hemorrhage were 100.4, 324.8, and 25.6 per 100,000 person-years in men, and 63.0, 154.5, and 54.4 per 100,000 person-years in women, respectively. After adjustment for age by using the direct method, the rates of stroke were 311.5 and 221.0 per 100,000 person-years in men and women, respectively (Table 2).

**Table 2.** Number of cases and incidence of stroke subtypes in men and women.

	Age (y)					Total	
	
	≤39	40-49	50-59	60-69	≥70	Crude	Age-adjusted*
	Men						

n	493	1,060	1,128	1,827	299	4,807	
Mean follow-up (y)	10.7	11.1	11.0	10.2	9.0	10.6	
Follow-up (person-years)	5,255	11,782	12,380	18,689	2,690	50,796	
No. of cases							
All strokes	1	15	33	143	37	229	
Cerebral hemorrhage	0	4	8	33	6	51	
Cerebral infarction	1	9	22	105	28	165	
Subarachnoid hemorrhage	0	2	3	5	3	13	
Not confirmed	0	0	0	0	0	0	

Incidence rate (/100,000 person-years)							
All strokes	19.0	127.3	266.6	765.2	1375.5	450.8	311.5
Cerebral hemorrhage	0	34.0	64.6	176.6	223.0	100.4	65.6
Cerebral infarction	19.0	76.4	177.7	561.8	1040.9	324.8	225.8
Subarachnoid hemorrhage	0	17.0	24.2	26.8	111.5	25.6	18.7
Not confirmed	0	0	0	0	0	0	

	Women						

n	677	1,536	2,108	2,777	371	7,469	
Mean follow-up (y)	10.4	11.1	11.1	10.8	9.6	10.8	
Follow-up (person-years)	7,021	16,985	23,375	29,965	3,576	80,922	
No. of cases							
All strokes	1	7	43	126	44	221	
Cerebral hemorrhage	1	1	10	33	6	51	
Cerebral infarction	0	3	13	76	33	125	
Subarachnoid hemorrhage	0	3	20	17	4	44	
Not confirmed	0	0	0	0	1	1	

Incidence rate (/100,000 person-years)							
All strokes	14.2	41.2	184.0	420.5	1230.4	273.1	221.0
Cerebral hemorrhage	14.2	5.9	42.8	110.1	167.8	63.0	39.4
Cerebral infarction	0	17.7	55.6	253.6	922.8	154.5	136.1
Subarachnoid hemorrhage	0	17.7	85.6	56.7	111.9	54.4	38.8
Not confirmed	0	0	0	0	28.0	1.2	

*: Adjusted for age by using the direct method

The incidence rate of MI was 2.7 times higher in men than in women after the adjustment for age (Table 3).

**Table 3.** Number of cases and incidence of myocardial infarction in men and women.

	Age (y)					Total	
	
	<39	40-49	50-59	60-69	>70	Crude	Age-adjusted*
	Men						

n	493	1,062	1,130	1,842	302	4,829	
Mean follow-up (y)	10.6	11.2	11.0	10.5	9.4	10.7	
Follow-up (person-years)	5,249	11,875	12,466	19,300	2,828	51,719	

Myocardial infarction	1	2	10	39	12	64	
Incidence rate (/100,000 person-years)	19.1	16.8	80.2	202.1	424.3	123.7	83.2

	Women						

n	677	1,539	2,110	2,796	372	7,494	
Mean follow-up (y)	10.4	11.1	11.2	10.9	9.9	10.9	
Follow-up (person-years)	7,021	17,051	23,531	30,544	3,685	81,832	

Myocardial infarction	0	0	6	14	8	28	
Incidence rate (/100,000 person-years)	0.0	0.0	25.5	45.8	217.1	34.2	30.9

*: Adjusted for age by using the direct method

## DISCUSSION

The JMS Cohort Study is a multicenter population-based cohort study on the incidence of newly diagnosed stroke and MI. In the past few decades, rapid westernization of lifestyle has occurred in Japan. Cholesterol intake in Japan has risen dramatically since the 1960s, and the levels of serum cholesterol are now comparable to those in the United States.^17^^,^^23^^,^^24^ The prevalence of hypertension has declined and is now slightly lower in Japan than in the US.^17^^,^^25^^,^^26^ While the proportion of obesity has increased in some segments of the Japanese population, it remains far lower than that seen in the US.^27^^,^^28^ On the other hand, the prevalence of smoking remains high among Japanese men. Data from the current study indicate the incidence rates of stroke, which appear to be lower than those reported previously, while the rates of MI appear to be stable.

In the present study, age-adjusted annual incidence rates of stroke for the total study period were 312 and 221 per 100,000 person-years in men and women, respectively. Some studies reported that in the past few decades, the incidence rate of stroke declined in Japanese cohorts starting between the 1960s and 70s.^16^^-^^19^^,^^29^

In the Hisayama study, a rapid decline in the stroke incidence rate was observed over 2 decades. In the 1960s, the age-adjusted annual incidence rates of all strokes were approximately 1200 and 600 per 100,000 person-years in men and women, respectively. The incidence rate of all strokes in the third cohort of the Hisayama study, starting in 1988, was 529 in men and 388 in women. These values are higher than those in the present study.^19^ Similar trends of stroke incidence were shown in some other Japanese cohort studies.^17^^,^^18^^,^^30^ The decline in stroke incidence was mainly due to a decline in the occurrence of cerebral hemorrhage. Blood pressure control and nutritional improvement contributed to this phenomenon.^17^^,^^19^

In Shiga Prefecture, around 1990, the age-adjusted incidence rates of stroke (per 100,000 person-years) were determined to be 269 in men and 168 in women after a 4-year follow-up.^31^ In a cohort of urban workers, the incidence rate of stroke was considerably lower than that observed in other studies, perhaps due to a healthy worker effect.^32^ The incidence of stroke was higher in men than in women in all cohort studies mentioned above, and the results of the present study were consistent with those of the previous studies.

In the US, the trend of stroke incidence was reported in the Framingham study; the incidence of stroke in the 1960 and 1970 cohorts was about half of that in the 1950 cohort. The crude annual incidence rate was approximately 200 per 100,000 person-years in the 1970 cohort.^8^ The incidence rate of stroke was lower in Western countries than that reported in the present study. In the US, the annual incidence rates of stroke were between 93 and 178 per 100,000 person-years in white men.^11^^,^^13^ In Europe, the MONICA Project showed that the annual incidence rates (per 100,000 person-years) of stroke were between 100 (Friuli, Italy) and 290 (Kuopio, Finland) in the case of men and between 60 (Friuli, Italy) and 190 (Novosibirsk, Russia) in the case of women. In half of the populations, the stroke incidence was twice as high in men as in women.^33^ However, another collaborative study showed that annual stroke incidence rates were between approximately 300 and 500 per 100,000 person-years, which was similar to our results. Sudlow et al^10^ revealed the following limitations of the MONICA Project: many parts of the world were not represented, many centers had participants aged 65 years or younger, and many centers used semi-retrospective methods.

In the Ni-Hon-San study conducted in the 1970s, Japanese men living in Japan were found to have a considerably higher stroke incidence rate than that of Japanese men living in Hawaii.^34^ Interestingly, the incidence rate of stroke in the Japanese-Hawaiians 35 years ago was similar to those seen in the data reported here. These results showed that environmental factors like lifestyle, including food, were likely to be strong contributors to the difference.

Previous studies in Japan have reported the incidence rates of MI, which were lower than those for stroke. In the present study, the age-adjusted annual incidence rate (per 100,000 person-years) of MI was 80 in men and 30 in women. Despite the apparent increase in the risk for MI with westernization over the previous decades, the rates of MI do not appear to have increased to the degree they have in other Asian countries.^35^^,^^36^ In the Hisayama study in Japan, the age-adjusted incidence rate of CHD did not change significantly over more than 3 decades.^19^ This fact was observed among both sexes. Although the rates seen in the present study were relatively lower than those seen in other studies, the criteria used for diagnosis varied among studies, and the MONICA criteria used here include only definite cases of MI. In addition, differences were noted in the criteria for the study samples.

Annual CHD incidence rates (per 100,000 person-years) were between 200 and 500 in men and between 60 and 150 in women in Western countries.^8^^,^^12^ The highest incidence rate (per 100,000 person-years) of CHD in the case of men was in North Karelia, Finland (835) and that in the case of women was in Glasgow, United Kingdom (265). Robertson et al reported a significantly greater incidence of MI and death from CHD in male Japanese-Hawaiian residents as compared with that in Japanese men living in Japan.^2^ Furthermore, a substantially greater incidence of MI was seen in Japanese people living in California than in those living in Hawaii. The rate of MI incidence in Japanese people living in Japan was 1.4 (per 1,000 person-years), half that in Japanese people living in Hawaii and 3-fold lower than that in Japanese people living in California.^2^ Moreover, reports of the Honolulu Heart Program and the Hiroshima/Nagasaki study, a part of the Ni-Hon-San Study, showed that the incidence of MI had not changed for about 20 years in Hawaii or in Japan.^35^^,^^37^

In the present study, the incidence rate of stroke was approximately 4 times higher than that of MI among men and 7 times higher among women. In most Western countries, the incidence rate of MI was higher than that of stroke.^8^^,^^36^ The incidence rates of stroke and MI in the present study were lower than those seen in the third Hisayama cohort, which began in 1988.^19^ However, the participation rate might have contributed to this difference. Serum total cholesterol levels have been increasing in recent years in Japan. Thus, the incidence rate of MI may increase in the future, and it is important to monitor the future trends of cardiovascular diseases in Japan.

There were some limitations of the current study. Although the study subjects were selected from a population-based health check-up system, they were not selected at random. Thus, the subjects were rather healthier than the general population, and the proportion of subjects treated for hypertension, diabetes mellitus, or hyperlipidemia was low. We had reported the standardized mortality ratio (SMR) of the study areas previously, and the SMRs were approximately 0.7 in both men and women.^20^ The present study was carried out in primarily rural areas, and the data may not be generalizable to urban populations. However, the response and follow-up rates were quite high. In the Akabane study, the incidence of stroke in the non-responders was considerably higher than that in those who were examined in the following health check-up.^16^ Not all of the study subjects could be followed up. Although we used the annual health check-up examination system, only about 60% of the participants returned for check-ups every year. Mail and telephonic follow-up was attempted in non-responders, and medical records were also checked; thus, only 7 persons were lost to follow-up. The present study was carried out in a standardized fashion in 12 different areas all over Japan and included more than 12,000 men and women. Most cohort studies conducted in Japan include only 1 area.

In conclusion, the incidence rate of stroke remains high and is remarkably higher than that of MI in both men and women. The incidence rates of both stroke and MI were higher in men than in women. Stroke remains a greater burden to healthcare in Japan than MI, whose incidence has remained paradoxically low despite apparently worsening trends of serum cholesterol and persistently high rates of smoking in men. These patterns are unlike those seen in other developed countries. Further research into the underlying causes of these differences is indicated.

## References

[1] Health and Welfare Statistics Association. Vital Statistics of Japan, 2000, Vol. 3. Tokyo (Japan): Health and Welfare Statistics Association; 2000.

[2] Robertson TL, Kato H, Gordon T, Kagan A, Rhoads GG, Land CE, Epidemiologic studies of coronary heart disease and stroke in Japanese men living in Japan, Hawaii and California. Coronary heart disease risk factors in Japan and Hawaii. Am J Cardiol 1977;39:244-9. 10.1016/S0002-9149(77)80198-7 835483

[3] Menotti A, Keys A, Kromhout D, Blackburn H, Aravanis C, Bloemberg B, Inter-cohort differences in coronary heart disease mortality in the 25-year follow-up of the seven countries study. Eur J Epidemiol 1993;9:527-36. 10.1007/BF00209531 8307138

[4] Boysen G, Nyboe J, Appleyard M, Sorensen PS, Boas J, Somnier F, Stroke incidence and risk factors for stroke in Copenhagen, Denmark. Stroke 1988;19:1345-53. 10.1161/01.STR.19.11.1345 3188119

[5] Haheim LL, Holme I, Hjermann I, Leren P. Risk factors of stroke incidence and mortality. A 12-year follow-up of the Oslo Study. Stroke 1993;24:1484-9. 10.1161/01.STR.24.10.1484 8378951

[6] Monique Verschuren WM, Kromhout D. Total cholesterol concentration and mortality at a relatively young age: do men and women differ? BMJ 1995;311:779-83. 10.1136/bmj.311.7008.779 7580439 PMC2550788

[7] Cholesterol, diastolic blood pressure, and stroke: 13,000 strokes in 450,000 people in 45 prospective cohorts. Prospective studies collaboration. Lancet 1995;346:1647-53. 10.1016/S0140-6736(95)92836-7 8551820

[8] Sytkowski PA, D’Agostino RB, Belanger A, Kannel WB. Sex and time trends in cardiovascular disease incidence and mortality: the Framingham Heart Study, 1950-1989. Am J Epidemiol 1996;143:338-50. 10.1093/oxfordjournals.aje.a008748 8633618

[9] Brown RD, Whisnant JP, Sicks JD, O’Fallon WM, Wiebers DO. Stroke incidence, prevalence, and survival: secular trends in Rochester, Minnesota, through 1989. Stroke 1996;27:373-80. 8610298

[10] Sudlow CL, Warlow CP. Comparable studies of the incidence of stroke and its pathological types: results from an international collaboration. International Stroke Incidence Collaboration. Stroke 1997;28:491-9. 10.1161/01.STR.28.3.491 9056601

[11] Sacco RL, Boden-Albala B, Gan R, Chen X, Kargman DE, Shea S, Stroke incidence among white, black, and Hispanic residents of an urban community: the Northern Manhattan Stroke Study. Am J Epidemiol 1998;147:259-68. 10.1093/oxfordjournals.aje.a009445 9482500

[12] Tunstall-Pedoe H, Kuulasmaa K, Mahonen M, Tolonen H, Ruokokoski E, Amouyel P. Contribution of trends in survival and coronary-event rates to changes in coronary heart disease mortality: 10-year results from 37 WHO MONICA project populations. Monitoring trends and determinants in cardiovascular disease. Lancet 1999;353:1547-57. 10.1016/S0140-6736(99)04021-0 10334252

[13] Rosamond WD, Folsom AR, Chambless LE, Wang CH, McGovern PG, Howard G, Stroke incidence and survival among middle-aged adults: 9-year follow-up of the Atherosclerosis Risk in Communities (ARIC) cohort. Stroke 1999;30:736-43. 10.1161/01.STR.30.4.736 10187871

[14] Ohno Y, Tamakoshi A. Japan collaborative cohort study for evaluation of cancer risk sponsored by monbusho (JACC study). J Epidemiol 2001;11:144-50. 10.2188/jea.11.144 11512570 PMC11735075

[15] Watanabe S, Tsugane S, Sobue T, Konishi M, Baba S. Study design and organization of the JPHC study. Japan Public Health Center-based Prospective Study on Cancer and Cardiovascular Diseases. J Epidemiol 2001;11:S3-7. 10.2188/jea.11.6sup_3 11763137 PMC11858366

[16] Okada H, Horibe H, Yoshiyuki O, Hayakawa N, Aoki N. A prospective study of cerebrovascular disease in Japanese rural communities, Akabane and Asahi. Part 1: evaluation of risk factors in the occurrence of cerebral hemorrhage and thrombosis. Stroke 1976;7:599-607. 10.1161/01.STR.7.6.599 1006736

[17] Shimamoto T, Komachi Y, Inada H, Doi M, Iso H, Sato S, Trends for coronary heart disease and stroke and their risk factors in Japan. Circulation 1989;79:503-15. 10.1161/01.CIR.79.3.503 2783893

[18] Nakayama T, Date C, Yokoyama T, Yoshiike N, Yamaguchi M, Tanaka H. A 15.5-year follow-up study of stroke in a Japanese provincial city. The Shibata Study. Stroke 1997;28:45-52. 10.1161/01.STR.28.1.45 8996487

[19] Kubo M, Kiyohara Y, Kato I, Tanizaki Y, Arima H, Tanaka K, Trends in the incidence, mortality, and survival rate of cardiovascular disease in a Japanese community: the Hisayama study. Stroke 2003;34:2349-54. 10.1161/01.STR.0000090348.52943.A2 12958323

[20] Ishikawa S, Gotoh T, Nago N, Kayaba K; Jichi Medical School (JMS) Cohort Study Group. The Jichi Medical School (JMS) Cohort Study: design, baseline data and standardized mortality ratios. J Epidemiol 2002;12:408-17. 10.2188/jea.12.408 12462275 PMC10681813

[21] Adams HP Jr, Bendixen BH, Kappelle LJ, Biller J, Love BB, Gordon DL, Classification of subtype of acute ischemic stroke: Definitions for use in a multicenter clinical trial: TOAST: Trial of ORG 10172 in Acute Stroke Treatment. Stroke 1993;24:35-41. 10.1161/01.STR.24.1.35 7678184

[22] WHO MONICA Project Principal Investigators. The World Health Organization MONICA Project (monitoring trends and determinants in cardiovascular disease): a major international collaboration. J Clin Epidemiol 1988;41:105-14. 10.1016/0895-4356(88)90084-4 3335877

[23] Arai H, Yamamoto A, Matsuzawa Y, Saito Y, Yamada N, Oikawa S, Serum lipid survey and its recent trend in the general Japanese population in 2000. J Atheroscler Thromb 2005;12:98-106. 10.5551/jat.12.98 15942120

[24] Carroll MD, Lacher DA, Sorlie PD, Cleeman JI, Gordon DJ, Wolz M, Trends in serum lipids and lipoproteins of adults, 1960-2002. JAMA 2005;294:1773-81. 10.1001/jama.294.14.1773 16219880

[25] van den Hoogen PCW, Feskens EJM, Nagelkerke NJD, Menotti A, Nissinen A, Kromhout D, The relation between blood pressure and mortality due to coronary heart disease among men in different parts of the world. N Engl J Med 2000;342:1-8. 10.1056/NEJM200001063420101 10620642

[26] Stamler J, Elliott P, Dennis B, Dyer AR, Kesteloot H, Liu K, INTERMAP: background, aims, design, methods, and descriptive statistics (nondietary). J Hum Hypertens 2003;17:591-608. 10.1038/sj.jhh.1001603 13679950 PMC6660162

[27] Yoshiike N, Seino F, Tajima S, Arai Y, Kawano M, Furuhata T, Twenty-year changes in the prevalence of overweight in Japanese adults: the National Nutrition Survey 1976-95. Obes Rev 2002;3:183-90. 10.1046/j.1467-789X.2002.00070.x 12164470

[28] Flegal KM, Carroll MD, Ogden CL, Johnson CL. Prevalence and trends in obesity among US adults, 1999-2000. JAMA 2002;288:1723-7. 10.1001/jama.288.14.1723 12365955

[29] Kodama K. Stroke trends in Japan. Ann Epidemiol 1993;3:524-8. 10.1016/1047-2797(93)90109-H 8167830

[30] Komachi Y, Tanaka H, Shimamoto T, Handa K, Iida M, Isomura K, A collaborative study of stroke incidence in Japan: 1975-1979. Stroke 1984;15:28-36. 10.1161/01.STR.15.1.28 6695428

[31] Kita Y, Okayama A, Ueshima H, Wada M, Nozaki A, Choudhury SR, Stroke incidence and case fatality in Shiga, Japan 1989-1993. Int J Epidemiol 1999;28:1059-65. 10.1093/ije/28.6.1059 10661648

[32] Kitamura A, Iso H, Iida M, Naito Y, Sato S, Jacobs DR, Trends in the incidence of coronary heart disease and stroke and the prevalence of cardiovascular risk factors among Japanese men from 1963 to 1994. Am J Med 2002;112:104-9. 10.1016/S0002-9343(01)01053-1 11835947

[33] Stegmayr B, Asplund K, Kuulasmaa K, Rajakangas AM, Thorvaldsen P, Tuomilehto J. Stroke incidence and mortality correlated to stroke risk factors in the WHO MONICA Project. An ecological study of 18 populations. Stroke 1997;28:1367-74. 10.1161/01.STR.28.7.1367 9227685

[34] Takeya Y, Popper JS, Shimizu Y, Kato H, Rhoads GG, Kagan A. Epidemiologic studies of coronary heart disease and stroke in Japanese men living in Japan, Hawaii and California: incidence of stroke in Japan and Hawaii. Stroke 1984;15:15-23. 10.1161/01.STR.15.1.15 6695420

[35] Kodama K, Sasaki H, Shimizu Y. Trend of coronary heart disease and its relationship to risk factors in a Japanese population: a 26-year follow-up, Hiroshima/Nagasaki study. Jpn Circ J 1990;54:414-21. 10.1253/jcj.54.414 2398621

[36] Truelsen T, Mahonen M, Tolonen H, Asplund K, Bonita R, Vanuzzo D; WHO MONICA Project. Trends in stroke and coronary heart disease in the WHO MONICA Project. Stroke 2003;34:1346-52. 10.1161/01.STR.0000069724.36173.4D 12738889

[37] Reed D, Maclean C. The nineteen-year trends in CHD in the Honolulu Heart Program. Int J Epidemiol 1989;18:S82-7. 10.1093/ije/18.Supplement_1.S82 2807711

